# Advances in Personalized Oncology

**DOI:** 10.3390/cancers16162862

**Published:** 2024-08-16

**Authors:** Hiba Mechahougui, James Gutmans, Gina Colarusso, Roumaïssa Gouasmi, Alex Friedlaender

**Affiliations:** 1 Oncology Department, Geneva University Hospital (HUG), 1205 Geneva, Switzerland; hiba.mechahougui@hcuge.ch (H.M.);; 2 Cancer Research Center of Lyon, CNRS UMR5286, Inserm U1052, University of Lyon, 69100 Lyon, France; 3 Clinique Générale Beaulieu, 1206 Geneva, Switzerland

**Keywords:** precision medicine, oncogenic drivers, precision oncology, personalized medicine, oncogenic alterations, driver mutations

## Abstract

**Simple Summary:**

Advances in next-generation sequencing have initiated a paradigm shift in cancer treatment, transitioning from traditional, organ-specific protocols to precision medicine. However, interpreting the clinical implications of an ever-growing catalog of genetic mutations remains challenging. This review explores the direct connections between genetic alterations and targeted therapies, addressing key genetic factors, the challenges in clinical application and research, and strategies for maximizing the therapeutic impact of these treatments.

**Abstract:**

Advances in next-generation sequencing (NGS) have catalyzed a paradigm shift in cancer treatment, steering the focus from conventional, organ-specific protocols to precision medicine. Emerging targeted therapies offer a cutting-edge approach to cancer treatment, while companion diagnostics play an essential role in aligning therapeutic choices with specific molecular changes identified through NGS. Despite these advances, interpreting the clinical implications of a rapidly expanding catalog of genetic mutations remains a challenge. The selection of therapies in the presence of multiple mutations requires careful clinical judgment, supported by quality-centric genomic testing that emphasizes actionable mutations. Molecular tumor boards can play an increasing role in assimilating genomic data into clinical trials, thereby refining personalized treatment approaches and improving patient outcomes.

## 1. Introduction

The advancement in cancer genomics, particularly the widespread adoption of next-generation sequencing (NGS) in the past two decades, has been instrumental in identifying genetic mutations responsible for cancer. This has enabled the development of therapies that intervene in the resulting aberrant signaling pathways. NGS is now integral to cancer diagnostics, aiding in the determination of mutations for selecting targeted treatments, prognostication, and other clinical decisions. Unlike conventional chemotherapy that non-selectively targets rapidly dividing cells, targeted therapies act on specific proteins resulting from cancer-causing mutations, offering greater efficacy and fewer side effects.

A landmark in targeted therapy was the discovery of the BCR-ABL fusion gene in chronic myelogenous leukemia (CML) and the subsequent development of imatinib, a BCR-ABL inhibitor [1]. This success has led to the Food and Drug Administration (FDA) approval of various targeted therapies, with many others currently under investigation.

Mutations in oncogenes can result in a variant protein or cause protein activation that can be specifically targeted. While mutations that result in a distinct targetable protein are rarer, many therapies have been successful in targeting overactivated or amplified proteins due to their high expression levels or the cancer’s dependence on these proteins. Other mutations may not be directly targetable but serve as biomarkers to guide the use of alternative therapies [2,3].

This review focuses on the direct links between alterations and targeted therapies, examining the key genetic factors, the challenges in clinical application and research, and the strategies for optimizing the therapeutic impact of these precision medicines.

## 2. Genomics, Proteomics, and the Use of Next Generation Sequencing

Advancements in genomics have revolutionized the understanding of cancer’s complexity and the immune system’s role in the disease, prompting a transition from tumor-centric treatments to personalized therapies guided by specific molecular markers. Genomics serves as a cornerstone for precision medicine by pinpointing exact genetic anomalies driving disease progression, leading to treatments tailored to these unique changes. Beyond genomics, RNA and protein profiling have emerged as critical in influencing disease outcomes [2].

Proteins execute cellular signals and are pivotal in numerous biological functions. Protein assays, which quantify protein levels and activities, can unearth vital insights into a disease’s biology [3]. Nonetheless, genomic-based patient-drug matching has generally yielded better results than protein assays, possibly due to the latter’s technical constraints. Despite this, incorporating protein and transcript assessments with genomic data has proved invaluable, particularly as panels incorporating immune signatures from DNA, RNA, and proteins have gained clinical relevance, offering a more holistic view of a disease and informing therapeutic targeting.

Transcriptomics, the exploration of RNA transcripts, leverages high-throughput methods like microarrays and RNA sequencing to uncover disease causation and identify appropriate treatments. The WINTHER trial, a milestone in precision medicine [4], illustrated that transcriptomics can enhance patient–therapy matching in clinical settings. Nonetheless, applying transcriptomic biomarkers faces hurdles, including RNA instability in stored samples, the complexity of bioinformatic processing, and limited result reproducibility [5].

NGS has demonstrated that the genetic alterations in advanced cancers defy traditional organ-based classifications, with metastatic tumors exhibiting distinct genomic and immune signatures that underscore the necessity for individualized genomic-based treatment strategies. Furthermore, recent declines in sequencing costs and durations, propelled by technological advancements, have expanded access for researchers and clinicians, enabling wider application in patient care.

## 3. Molecular Targets and Mechanisms of Action

### 3.1. Mitotic Cycle and DNA Repair Targeting

Aberrations in the cell **cycle** can lead to unrestrained cell growth, a fundamental characteristic of cancer. Cell cycle progression is governed by cyclin-dependent kinases (CDKs), which depend on cyclin proteins for their activation and phosphorylation. Notably, CDK4 and CDK6 are integral in regulating growth signals and mediating the cell cycle’s transition from the G1 to the S phase. This regulation involves D-type cyclins, which activate CDK4 and CDK6, leading to phosphorylation of the retinoblastoma protein 1 (RB1) and subsequent release of E2F transcription factors, which are key to cell cycle progression. Given the pivotal role of the CDK4/6-RB1 pathway in cell proliferation and cancer development, targeting CDK4/6 offers a valuable cancer treatment strategy, potentially arresting the cell cycle in the G1 phase and reducing cell survival. CDK4 and CDK6 have identical functions, hence simultaneous inhibition is critical to counterbalance any compensatory mechanisms. Cyclin D, a key target for estrogen receptor-mediated transcription, initiates the CDK4/6-RB1 pathway, particularly implicating its dysregulation in hormone receptor-positive breast cancers. Currently, three CDK inhibitors, palbociclib, abemaciclib, and ribociclib are approved for clinical use [6].

**Mutations in the breast cancer 1 (BRCA1) gene**, crucial for DNA repair, can be inherited (germline) or acquired (somatic). Independent studies have found that somatic mutations in the BRCA1 gene comprise a significant portion of mutations in patients with ovarian cancer (OC), occurring in about 5–7% of cases. These somatic mutations are part of the “BRCAness” concept, where no germline mutations of BRCA1 or BRCA2 are found, yet a DNA repair deficiency arises due to issues in homologous recombination. There is no noticeable difference in the progression or severity of OC between patients with somatic or germline BRCA1 mutations. Both groups exhibit heightened responsiveness to platinum-based chemotherapies and olaparib, a poly ADP-ribose polymerase (**PARP) inhibitor**. PARP inhibitors work by binding to PARP1, a protein involved in DNA damage detection and repair facilitation. These inhibitors impede DNA repair, causing death in cells that cannot rely on homologous recombination due to BRCA1/2 mutations. The effectiveness of PARP inhibitors, like olaparib and rucaparib, is partly attributed to their ability to “trap” PARP on DNA, beyond mere catalytic inhibition. Even patients without BRCA mutations but with faults in other homologous recombination-related genes have shown positive responses to these treatments. Nonetheless, resistance to PARP inhibitors can develop through mechanisms that either diminish PARP trapping or restore the homologous recombination repair pathways [7,8].

The **P53 protein**, often called the “guardian of the genome,” plays a crucial role in maintaining genetic stability by preventing the transmission of genetic mutations to progeny cells. Cells devoid of p53 retain some checkpoint and DNA repair capabilities, but cells with mutant p53 proteins exhibit significantly increased genomic instability, such as interchromosomal translocations and aneuploidy, which highlights the gain-of-function (GOF) activity of p53 mutants. These mutants interfere with the initial stages of DNA double-strand break repair, disrupting the MRE11-RAD50-NBS1 (MRN) complex’s recruitment, which in turn hinders the activation of ATM, a key sensor of DNA damage, leading to compromised cell cycle checkpoint integrity. Mutant p53 also disrupts the DNA replication process, further contributing to genomic instability. Mutant p53 proteins are also known to drive the limitless replication and insensitivity to growth inhibitors observed in cancer cells, aiding in malignant transformation. Through interactions with cell cycle regulators and transcription factors, mutant p53 enhances the expression of genes that promote cell proliferation and survival. This includes interactions that promote cell cycle progression and the stabilization of replication forks, facilitating the duplication of abnormal DNA. In terms of metastases, mutant p53 proteins upregulate key EMT-related transcription factors and interact with various molecular pathways to enhance cell migration and invasion capabilities. They also contribute to forming and maintaining cancer stem cells (CSCs), which are often associated with increased resistance to chemotherapy and radiotherapy. Mutant p53 proteins regulate the expression of chemo- and radio-resistance genes, such as MDR1 and NRF2, and can interfere with apoptosis by inhibiting caspase-9 and the p63/73-dependent induction of pro-apoptotic proteins. Mutant p53 proteins also play a role in shaping a pro-oncogenic tumor microenvironment by inducing the secretion of pro-inflammatory cytokines and angiogenesis, which supports tumor progression and chemoresistance. They modulate the extracellular matrix (ECM) and interact with key pathways like NF-κB to promote tumor development and migration. Mutant p53 inhibitors, including MK-1775, COTI-2, APR-246, Arsenic Trioxide, and ReACp53, are under investigation. Of these, APR-246, also known as Eprenetapopt, has progressed the furthest in clinical trials, being the only one to reach phase III. Eprenetapopt, a methylated derivative of PRIMA-1 with improved membrane permeability, works by converting mutant p53 back to a wild-type conformation. Although its full reactivation potential is not fully declared, it has demonstrated p53-dependent biological effects in patients with mutant p53 [9,10].

The pursuit of therapies that target tumor suppressor gene mutations is complicated, and finding broadly applicable approaches for the diverse range of tumor suppressor mutations is an ongoing challenge that will require further research and innovation.

### 3.2. Monoclonal Antibodies

Monoclonal antibodies (mAbs) are synthetic proteins designed to mimic the immune system’s ability to combat harmful antigens. They are especially potent in oncology, where they target specific molecules such as cancer cell receptors or the extracellular ligands that activate them. By binding to these targets, mAbs can disrupt vital growth signals, leading to cancer cell death. This targeted approach is exemplified by the use of trastuzumab in **HER2-positive** breast cancer, where it effectively hinders the proliferation of cancer cells by blocking HER2 receptors. Pertuzumab complements this action by targeting a different HER2 site, disrupting its interaction with HER3 and enhancing the overall therapeutic effect when used in combination with trastuzumab [11,12]. Further refining this strategy, mAbs such as margetuximab have been developed to enhance immune cell recruitment, offering potential benefits even after resistance to other mAbs has set in. The advancement of antibody-drug conjugates, like trastuzumab-emtansine, marks a significant leap by delivering cytotoxic drugs directly to cancer cells [13].

Bevacizumab, another mAb, targets vascular endothelial growth factor (**VEGF)-A**, thereby inhibiting the VEGF-A/VEGF receptor interaction and the pathways leading to new blood vessel formation. It has shown efficacy across a range of cancers, including colorectal and lung cancers, when used alongside chemotherapy [14].

**The epithelial growth factor receptor (EGFR) family**, with its four members, plays a pivotal role in cell survival by initiating intracellular signaling upon ligand binding. Overexpression or abnormal expression of these receptors can lead to cancer progression, with ErbB2 (HER2/neu) being notably implicated in many cancers. Monoclonal antibodies against these receptors can reduce toxicity to normal cells due to their specificity. EGFR-directed therapy with agents like cetuximab and panitumumab is a cornerstone in treating colorectal cancers with a wild-type EGFR status, as mutations in the EGFR pathway, such as RAS mutations, can negate the benefits of such treatments [15].

**Claudin 18** is crucial for the cohesion of epithelial cells in living organisms and is fundamental to maintaining a barrier function. The claudin (CLDN) family consists of at least 27 proteins that span the membrane. It is hypothesized that the variety of claudins across different tissues provides specialized barrier functions unique to each organ. In addition to their role in cell adhesion, CLDNs are involved in various cellular signaling pathways and cancer-related processes including inflammation, cell growth and survival, tumor spread, the transformation of epithelial cells to a mesenchymal state (EMT), metastases, and the maintenance of cancer stem cells within tumors. For example, reports have shown that CLDN2, CLDN7, and CLDN9 contribute to the enhanced invasion and migration of gastric cancer (GC) cells. CLDN6 has been linked to the induction of EMT via the LATS1/2-YAP1 pathway in GC tissues. CLDN18 is predominantly found in the lungs and stomach. There are two main splice variants of the CLDN18 gene: CLDN18.1 and CLDN18.2. CLDN18.1 is mainly located in the alveolar epithelium of the lungs, with lower expression in the airway epithelial cells, and absent in the endothelial junctions of the lungs, where it plays a role in maintaining the equilibrium of the alveolar barrier. The CLDN18.2 variant is part of the tight junction complex in both normal and cancerous gastric epithelial cells, first identified by Tsukita S. in 1998. Tight junctions are critical for cell–cell adhesion and serve as barriers that regulate the diffusion of solutes through cell gaps, paracellular transport, signal transduction, and interaction with the cytoskeleton. In healthy gastric mucosal tissue, CLDN18.2 is exclusively present in the differentiated cells of the gastric mucosa, excluding the gastric stem cell region. However, in various malignant tumors—including those of the stomach, gastroesophageal junction (GEJ), esophagus, colon, pancreas, liver, breast, ovary, and non-small cell lung—it is widely present. In normal cells, the CLDN18.2 component of the adhesion complex is inaccessible, but malignant transformation disrupts cell adhesion and makes the CLDN18.2 accessible on the surface of gastric adenocarcinoma cells, presenting it as a viable therapeutic target. CLDN18.2-positive GC is more frequently seen in signet-ring cell carcinoma and diffuse GC, which are associated with the genomically stable category of gastric cancers, as classified by the Cancer Genome Atlas Network Research. This type of tumor typically presents with a diffuse pathologic subtype and appears at a younger age [16,17].

**Dual EGFR and MET inhibition**. In EGFR-mutated non-small cell lung cancer (NSCLC), resistance to EGFR tyrosine kinase inhibitors (TKIs) becomes inevitable over time. Resistance mechanisms to modern TKIs often involve changes in the MET pathway, such as amplifications and mutations. MET amplification, detected in a subset of patients treated with the EGFR TKI osimertinib, and increased levels of the MET ligand, represents around 15% of EGFR TKI resistance [18]. Phase II data reveal activity of combining tepotinib, a MET inhibitor, with Osimertinib upon progression on osimertinib with MET amplification [19]. Resistance to MET TKIs can also occur through the upregulation of the EGFR pathway. The interaction between EGFR and MET, including the formation of complexes that drive oncogenic signaling, underscores the potential of dual inhibition as a therapeutic strategy. Amivantamab, a bispecific antibody targeting both EGFR and MET, has been developed to inhibit these pathways simultaneously, aiming to overcome resistance mechanisms and improve treatment outcomes for patients with NSCLC [20].

Despite the effectiveness of mAbs, the development of resistance through immune evasion, receptor domain changes, or alternative pathway activation remains a challenge. The future of cancer treatment with mAbs is promising yet complex, requiring a balance of targeted strategies and a deep understanding of the underlying mechanisms of cancer cell survival and resistance. This approach necessitates continuous innovation and precision to adapt to the dynamic nature of cancer, ensuring the most effective treatments reach the patients who need them.

### 3.3. Tyrosine Kinase Receptor Inhibitors

The discovery of imatinib sparked significant interest in targeting tyrosine kinases mutated in various cancers [21]. Advances in sequencing have identified numerous potential targets, and developments in chemistry have increased the number of these targets deemed “druggable.” Tyrosine kinases, which generally function as growth factor receptors or in direct interaction with them, and serine/threonine kinases, which respond to a broad range of cellular signals, can be inhibited by several mechanisms. These include competing for the ATP-binding site in their active or inactive conformations, allosteric inhibition, or other methods. Monoclonal antibodies targeting the extracellular domains of tyrosine kinases have become a valuable therapeutic strategy [22].

Drugs targeting the EGFR/HER family of receptor tyrosine kinases were among the first groups of targeted therapies FDA-approved. Mutations in these kinases led to uncontrolled signaling in various cancers, including non-small cell lung cancer and breast cancer [6]. While first-generation EGFR inhibitors were effective, resistance often developed, leading to the creation of more selective inhibitors like osimertinib [23].

Targeting fibroblast growth factor receptors (FGFRs), platelet-derived growth factor receptors (PDGFRs), and vascular endothelial growth factor receptor (VEGFR) family kinases has also shown clinical promise. These pathways typically involve angiogenesis, and while multikinase inhibitors have shown modest clinical success, more specific FGFR inhibitors are under investigation [24].

The therapeutic landscape has been enriched by TKIs treating cancers with mutations in kinases like JAK2, FLT3, ALK, MET, NTRK, RET, and ROS1. However, resistance to targeted therapy remains an issue, typically due to mutations that hinder drug binding or the activation of compensatory signaling pathways [6].

Multi-targeted kinase inhibitors have been both a boon and a challenge in cancer therapy. Their efficacy in certain contexts contrasts with the difficulties in pinpointing the specific targets responsible for therapeutic benefits. Ongoing research aims to understand the detailed mechanisms of TKI response and resistance to inform the development of the next generation of more effective inhibitors [25].

### 3.4. Downstream Signaling Effectors

**Figure 1:**
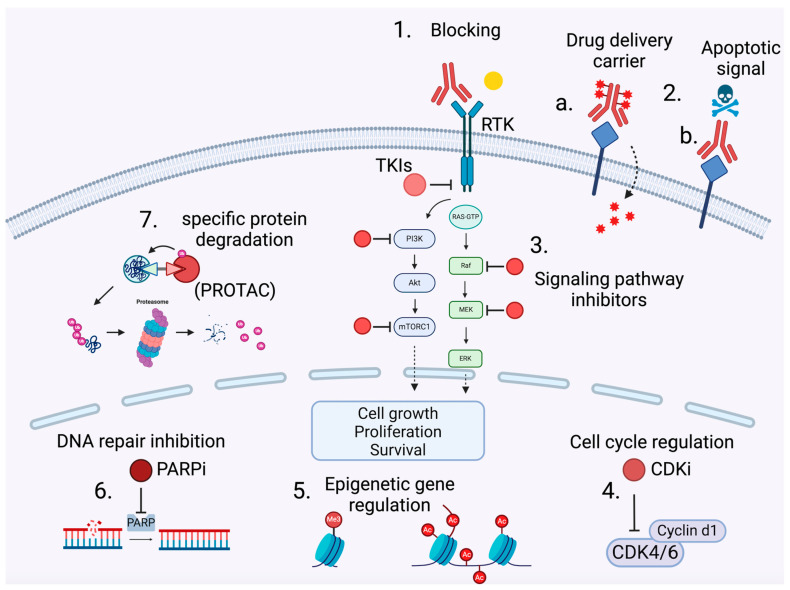
Therapeutic targets and mechanisms of action in precision medicine. Made with BioRender. **1. Antibodies** (in red) bind to specific receptors on the cell membrane, initiating or inhibiting various signaling pathways. **2. Cell death mechanisms**: (a) The antibody is linked to a drug conjugate, which can be a chemotherapeutic agent, a radioactive substance, or a toxin. The conjugation allows the drug to be delivered directly to the target cells. (b) Antibody binding to certain receptors can lead to programmed cell death (apoptosis). **3. Small molecules**, such as tyrosine kinase inhibitors, can inhibit the activation of proliferative signaling pathways such as the PI3K/Akt/mTOR and RAS/RAF/MEK/ERK pathways. **4. Activation of the PI3K/Akt/mTOR and RAS/RAF/MEK/ERK pathways** leads to the expression of Cyclin D1, which forms a complex with CDK4/6 (cyclin-dependent kinase 4/6), regulating cell cycle progression. CDK4/6 inhibitors are currently used as the standard of care in breast tumors. **5. Histones** are modified by acetylation (Ac) and methylation (Me3), influencing gene transcription. To compensate for identified chromatin modifier mutations, the potential of targeting enzymes like DNA methyltransferases and histone deacetylases is currently being evaluated. **6. PARP (Poly(ADP-ribose) polymerase)**, involved in DNA repair in response to damage, is targeted by PARP inhibitors. **7. The PROTAC strategy**: proteins marked for degradation are directed to the proteasome, where they are broken down into peptides.

**RAS-RAF-MEK pathways.** In the cascade of cellular signaling, tyrosine kinases initiate the relay of extracellular signals to intracellular serine/threonine kinases and other crucial proteins, such as RAS. These mutations can hijack the signaling pathways and promote cancer development. Targeting strategies similar to those used for tyrosine kinases have been applied to serine/threonine kinases, while monoclonal antibodies are effective only against those with extracellular domains [26]. The MAPK pathway, frequently mutated in cancers, includes RAS GTPases and RAF/MEK/ERK serine/threonine kinases. RAS, a GTPase, transitions between inactive and active states and, upon activation, triggers a signaling cascade involving RAF, MEK1/2, and ERK1/2, leading to changes in gene expression and cell behavior. Mutations in A-RAF, B-RAF and C-RAF, MEK1/2, and RAS are common, with BRAF (mainly V600) mutations, being able to signal independently. Early RAF inhibitors like sorafenib had limited efficacy [27], but newer inhibitors like vemurafenib, dabrafenib, and encorafenib have shown higher potency against the monomeric V600E-mutant BRAF protein [28,29]. Mutations in the RAS family, genes NRAS, KRAS, and HRAS are prevalent across various cancers. Historically considered “undruggable”, recent efforts have shifted toward directly targeting RAS-mutant proteins. Mutations typically occur at G12, G13, and Q61, with KRASG12C amenable to targeting due to its unique structure. Allosteric inhibitors like sotorasib and adagrasib stabilize the GDP-inactive state of RAS, though resistance can still develop. Other RAS mutations remain challenging targets due to high intrinsic GTP hydrolysis rates and the absence of a reactive cysteine residue [30] (Figure 1).

**PI3K-Akt-mTOR.** The PI3K/AKT/mTOR pathway is another critical signaling route often mutated in cancer. PI3K phosphorylates PIP2 to form PIP3, recruiting AKT for activation, which in turn activates mTOR and other downstream regulators. This pathway is usually regulated by PTEN, which dephosphorylates PIP3. Mutations and amplifications in PI3K, AKT, and mTOR, along with PTEN inactivation, are common. PI3K inhibitors have seen the most use, with several being FDA-approved, including alpelisib, which specifically targets PIK3CA-mutant breast cancer. AKT inhibitors are still under investigation, and mTOR inhibitors have been approved for various indications, though not specifically for genetically defined cancers [31]. Serine/threonine kinase inhibitors often exhibit limited clinical success, and patient selection based on tumor genetics is complex. Successes include RAF and MEK inhibitors for BRAF V600 mutant tumors and KRAS G12C inhibitors [32] (Figure 1).

### 3.5. Epigenetic Targets

Epigenetics is a field of genetics focused on studying heritable changes in gene expression that do not involve alterations to the underlying DNA sequence. These changes are tightly controlled by various chemical modifying enzymes and proteins that recognize specific modifications.

**EZH2**. Targeting chromatin modifier mutations has become a promising approach in cancer therapy, with mutations in these genes being prevalent across various cancers. The majority of FDA-approved drugs for chromatin modifiers are not based on genetically defined populations but have shown efficacy in certain cancers by targeting enzymes like DNA methyltransferases and histone deacetylases [33]. EZH2, a histone methyltransferase, has also been targeted successfully, particularly in B cell lymphomas where gain-of-function mutations lead to sustained proliferation. Tazemetostat, an EZH2 inhibitor, has shown efficacy in EZH2-mutant diseases and some benefit in non-mutant diseases, suggesting a broader therapeutic potential [34] (Figure 1).

**IDH1/2 mutations**, often seen in acute myeloid leukemia, cholangiocarcinoma, and gliomas, produce an oncometabolite that disrupts DNA methylation and differentiation. Inhibitors targeting IDH1/2 have been effective in reducing this oncometabolite and promoting differentiation and death in cancer cells with these mutations [35]. As the understanding of the roles of chromatin modifiers in cancer progresses, targeting specific mutations in these genes is increasingly viable. While early mutations in chromatin modifiers like IDH1/2 are pivotal in glioma development, their roles may evolve from driving to secondary as the cancer progresses, challenging the effectiveness of targeting these mutations [36]. The complexity of targeting mutations at different stages of cancer progression, not just for chromatin modifiers but across various gene classes, will require extensive research to fully understand and develop effective therapies.

**HDAC inhibitors.** Histone deacetylases (HDACs) are key epigenetic enzymes that regulate gene expression by removing acetyl groups from histone proteins, and they have been implicated in various cancers [37]. Abnormal HDAC activity can alter the expression of genes involved in critical cancer-related processes like cell growth and death, leading to potential targets for cancer therapy. Several HDAC inhibitors have received FDA approval, such as vorinostat for cutaneous T-cell lymphoma [38]. HDAC inhibitors as monotherapy have shown promise primarily in hematological cancers, but limited efficacy in solid tumors, possibly due to its non-specific effects on angiogenesis and immune cell function, alongside pharmacokinetic challenges [6].

### 3.6. Hedgehog Pathway Inhibitors

The hedgehog signaling pathway is a vital regulator in embryonic development and tissue repair that can be triggered by the interaction of HH ligands with the Patched-1 receptor. When HH ligands are not present, Patched-1 inhibits the signaling pathway by restraining Smoothened, a transmembrane protein involved in the pathway. The binding of the ligands to Patched-1 leads to its internalization and degradation, allowing Smoothened to accumulate in the primary cilium and become phosphorylated. This active Smoothened then facilitates the activation of GLI transcription factors, which stimulate the expression of genes responsible for cell growth, survival, and differentiation. Abnormal activation of the hedgehog pathway can contribute to the development and progression of various types of cancers, including both solid and hematological malignancies, and is also implicated in the maintenance of cancer stem cells. These aberrations can occur through overexpression of hedgehog ligands or mutations in Patched-1 or Smoothened [39]. Given its significant role in cancer, the hedgehog pathway represents a promising target for oncological therapies. Vismodegib is an orally administered inhibitor that targets the Smoothened receptor within the hedgehog signaling pathway, and is now validated in the treatment of advanced stages of basal cell carcinoma [40].

### 3.7. Circulating Tumor Markers, ctDNA

Circulating tumor DNA (ctDNA) testing is a minimally invasive technique increasingly adopted for guiding anticancer therapy selection and tracking treatment efficacy. It involves analyzing DNA fragments released into the bloodstream from tumors. CtDNA offers a holistic view of a tumor’s genetic diversity, surpassing the limited snapshot provided by a single biopsy, which is particularly valuable when dealing with tumor heterogeneity [41,42]. Moreover, ctDNA levels in the blood can indicate treatment effectiveness, revealing the changing landscape of tumor subclones, thereby informing therapeutic adjustments. This aspect of ctDNA testing offers critical insights into the tumor’s evolutionary dynamics and aids in fine-tuning treatment plans. Circulating tumor cells (CTCs), shed from primary tumors into the bloodstream, are associated with worse outcomes in various cancers, such as breast cancer and hormone-resistant prostate cancer. The number of CTCs present in the blood is a potential indicator of progression-free and overall survival, especially in metastatic breast cancer [43].

## 4. Selective Examples of Targeted Therapies in Cancers

### 4.1. Targeted Therapies in Breast Cancer

#### 4.1.1. HER2 Targeting Strategy

HER2-positive breast cancers, representing about 15–20% of all breast cancer cases, are known for their aggressive nature and poorer prognosis compared to HER2-negative tumors [44]. Advances in targeted therapies have, however, significantly improved outcomes for patients with this subtype [45]. 

The CLEOPATRA trial was a milestone in establishing first-line treatment protocols. It demonstrated that adding pertuzumab to docetaxel and trastuzumab significantly increased median progression free survival (mPFS) from 12.4 months to 18.5 months [46]. This combination also led to a 16.3-month improvement in median overall survival (mOS) after more than eight years of follow-up [47]. In second-line therapy, the EMILIA [48] and TH3RESA [49] trials initially established ado-trastuzumab emtansine (T-DM1) as the standard, based on consistent PFS and OS data. However, the DESTINY-Breast03 trial [50] later showed that trastuzumab deruxtecan significantly improved PFS compared with T-DM1, with a 12-month PFS rate of 75.8% versus 34.1% for T-DM1. The objective response rate (ORR) was 79.7% with trastuzumab deruxtecan, as opposed to 34.2% with T-DM1. The HER2CLIMB trial [51] underscored the effectiveness of tucatinib, a HER2-selective tyrosine kinase inhibitor, combined with capecitabine and trastuzumab, especially for patients with brain metastases. This supports its consideration as a second-line option for such patients. Lapatinib, when combined with trastuzumab, has shown improved PFS in TKI-naive patients [52], while neratinib, an irreversible pan-HER TKI, demonstrated a modest improvement in PFS in the NALA study [53]. Margetuximab-cmkb, evaluated in the SOPHIA trial, improved PFS to 5.8 months compared to 4.9 months with trastuzumab [54]. The prevalence of HER2 mutations and the aggressive nature of HER2-positive breast cancers highlight the significance of these targeted therapies. The improvements in treatment have not only enhanced survival rates but also offered a better quality of life for patients with this challenging breast cancer subtype.

#### 4.1.2. CDK4/6 Targeting Strategy

For first-line treatment of ER-positive, HER2-negative metastatic breast cancer (MBC), CDK4/6 inhibitors combined with endocrine therapy (ET) are regarded as the standard of care. This combination has shown improved PFS and OS with a favorable toxicity profile in various trials, scoring 3–5 on the ESMO-Magnitude of Clinical Benefit Scale (ESMO-MCBS) v1.1 [45]. ET plus CDK4/6 inhibition has been found to be as effective, if not more so, than chemotherapy (ChT) with less toxicity [55,56], making it the preferred choice unless there is immediate risk of organ failure. The possibility of rechallenging with CDK4/6 inhibitors after progression on them exists, especially after a treatment-free interval of at least 12 months, although data are limited [45]. These inhibitors are beneficial in both de novo and recurrent MBC, regardless of primary or secondary endocrine resistance, and are effective in postmenopausal or premenopausal women (the latter requiring a luteinizing hormone-releasing hormone agonist) and in men. For patients not relapsing on an aromatase inhibitor (AI), or within 12 months after stopping adjuvant AI, combining a CDK4/6 inhibitor with an AI is recommended. All three approved CDK4/6 inhibitors—palbociclib, ribociclib, and abemaciclib—appear similarly effective in the metastatic setting, though direct comparisons are not feasible due to diverse inclusion criteria in trials [45]. Palbociclib and ribociclib require combination with ET for efficacy, unlike abemaciclib, which has some single-agent efficacy [57]. Palbociclib, in combination with letrozole, has significantly extended PFS by 24.8 months, as demonstrated in the PALOMA-2 trial [58]. Ribociclib, also used alongside letrozole, has shown remarkable efficacy by improving PFS to about 25.3 months compared to 16.0 months with placebo, according to the MONALEESA-2 trial [59]. Abemaciclib has further underscored the effectiveness of CDK4/6 inhibitors, improving mPFS by 9.4 months as a monotherapy and up to 16.4 months when used with fulvestrant, as evidenced by the MONARCH 2 trial [60].

#### 4.1.3. PIK3CA/AKT Inhibitors

In the second-line setting for ER-positive, HER2-negative MBC after progression on a CDK4/6 inhibitor, testing for somatic PIK3CA and ESR1 mutations is recommended, especially if further AI therapy is considered, as well as for germline BRCA1/2 and PALB2 mutations limited. The optimal sequence of endocrine-based therapy post-CDK4/6 inhibitor progression remains uncertain and depends on prior treatments, the duration of response to previous ET, disease burden, patient preference, and treatment availability [45]. Second-line options include fulvestrant-alpelisib for PIK3CA-mutated tumors. The SOLAR-1 phase III trial showed that alpelisib with fulvestrant offers a PFS benefit for patients previously treated with an AI, but increased toxicity, particularly hyperglycemia and gastrointestinal issues, was noted [61]. Furthermore, AKT inhibitors like capivasertib are undergoing rigorous investigation for their efficacy against breast cancers with specific genetic aberrations such as PIK3CA mutations or the AKT1E17K mutation. After the positive results of the CAPITELLO-291 trial, showing a doubling of PFS from 3.6 to 7.2 months, the role of capivasertib is being further explored in the FAKTION trial [62], examining its ability to improve PFS and overall survival when paired with other therapeutic agents.

##### PARP Inhibitors

PARP inhibitors such as olaparib and talazoparib have emerged as critical in the treatment of BRCA-mutated breast cancer. The OlympiAD trial [63] has highlighted olaparib’s ability to improve PFS by 2.8 months over standard therapy. Similarly, talazoparib has offered a mPFS advantage of 8.6 months over 5.6 months with the physician’s choice of treatment in the EMBRACA trial [64]. In gBRCAm carriers, PARP inhibitors offer improved PFS and QoL, but not OS compared to single-agent chemotherapy [65,66].

##### mTOR Inhibitors

Other evidence-based targeted therapies include exemestane-everolimus, tamoxifen-everolimus, fulvestrant-everolimus, and finally PARP inhibitors for tumors harboring germline BRCA mutations (gBRCAm). Indeed, the BOLERO-2 trial found that everolimus-exemestane improved mPFS by 5.3 months versus placebo-exemestane with no significant OS or quality-of-life benefit [67]. Prior exposure to CDK4/6 inhibitor therapy might not impact survival outcomes for patients receiving everolimus-exemestane [68]. Prophylaxis, such as dexamethasone oral solution, is recommended to prevent stomatitis with everolimus use [69]. The BOLERO-6 trial suggested a PFS benefit of exemestane-everolimus over everolimus alone, though the benefit might be overstated [70]. Third-line treatment and beyond should consider sensitivity to previous treatments, time to progression, gBRCAm status, tumor biology, and resistance mechanisms. For endocrine-sensitive patients, continuing ET with agents not used previously in the metastatic setting is an option to delay chemotherapy and achieve some clinical benefit [45].

### 4.2. Targeted Therapies in Cholangiocarcinoma

#### 4.2.1. FGFR Targeting Strategy

Molecular targets such as FGFR2 fusion, IDH1/2 mutation, and HER2 amplification are currently being evaluated for targeted treatment approaches in cholangiocarcinoma (CCA), with most of these strategies still in the clinical investigation phase [71]. In the context of FGFR, a study involving 115 patients with CCA showed that mutations in FGFR2 were the most frequent at 6.1%, in contrast to FGFR1 mutations at 0.9% and no mutations in FGFR3–5 [72,73,74]. Recent developments in tyrosine kinase inhibitors (TKIs) have shown effectiveness particularly for patients with FGFR2 fusions. The FDA has approved pemigatinib and infigratinib for treating CCA patients with FGFR2 fusions post-standard chemotherapy treatment [75]. Pemigatinib, targeting FGFR1/2/3, was the first of these to receive approval for advanced CCA treatment [76]. In the FighT-202 phase II multicenter trial, an overall response rate (ORR) of 35.5% was observed in patients with FGFR2 fusion/rearrangement and those with other FGF/FGFR alterations [73]. Infigratinib, an inhibitor of FGFR1/3, was tested on advanced iCCA patients with FGFR2 gene fusions resistant to standard chemotherapy, showing an ORR of 31.0% and a confirmed ORR (cORR) of 26.9%, with the most common severe adverse events being hypophosphatemia, hyperphosphatemia, and hyponatremia [77]. Another study confirmed infigratinib’s significant clinical activity and manageable toxicity in patients with FGFR2 fusion/mutation/amplification who were resistant to gemcitabine [78]. The PROOF 301 phase III trial is currently evaluating infigratinib as a first-line treatment for patients with FGFR2-positive advanced CCA [79].

Derazantinib, which inhibits FGFR and other kinases such as KIT, VEGFR1, and DDR, was assessed in a phase I/II trial involving 29 iCCA (intrahepatic CCA) patients who were chemotherapy-naive or resistant. The drug showed an overall survival (OS) of 20.7% and a disease control rate (DCR) of 82.8% [80]. Erdafitinib, an inhibitor of FGFR1/2/3/4, was examined in a phase IIa study in Asia with 22 CCA patients, resulting in an ORR of 40.9%, a mPFS of 5.6 months, and a mOS of 40.2 months [81]. Additionally, futibatinib, a selective and irreversible inhibitor of FGFR1/2/3/4, significantly improved clinical outcomes in advanced iCCA patients with FGFR2 gene fusion-rearrangement in a phase II study, following one or more prior lines of systemic therapy [82]. FGFR2 monotherapy is suggested to be more efficacious and less toxic compared to conventional chemotherapy and is considered a viable second-line therapy for terminal CCA. Management of FGFR inhibitor side effects includes dietary changes and phosphate-lowering therapies, alongside nutrition and sleep optimization, and managing diarrhea with fluid intake and probiotics [83].

#### 4.2.2. IDH1/2 Targeting Strategy

Regarding IDH1/2 mutations, these occur in about 20–25% of iCCA patients and are rare in other CCA subtypes. IDH1 and IDH2 play a role in DNA transcription and repair, typically converting isocitrate to a-ketoglutarate [71]. Mutations in these genes can lead to epigenetic changes such as increased production of 2-hydroxyglutarate, resulting in DNA damage and histone methylation. Research from 2019 found IDH1 mutations in 13% of iCCA patients and 0.8% in eCCA patients [84], with IDH-1 mutations more prevalent in non-hepatitis CCA patients [85]. The ClarIDHy phase III trial showed that ivosidenib, an inhibitor of IDH1/2, was more effective than a placebo in advanced CCA patients with IDH1/2 mutations, improving mPFS and mOS [86]. Consequently, ivosidenib has been approved by the FDA for use in chemotherapy-resistant iCCA patients with IDH1/2 mutations [87].

#### 4.2.3. RAS-MEK-ERK Targeting Strategy

Finally, the RAS-RAF-MEK–ERK pathway, often activated by mutations in KRAS found across all CCA subtypes, is associated with a poor prognosis. Despite a lack of effective RAS inhibitors, focus has shifted to inhibiting downstream components such as BRAF, especially the V600E mutation, present in 1–6% of CCA patients, predominantly iCCA^1^. A study indicated that this mutation was linked with advanced stages, chemoresistance, and reduced survival [88]. Combination therapies, like dabrafenib (a BRAF inhibitor) with trametinib (a MEK inhibitor), have shown promise in treating these mutations. A phase II trial of this combination in CCA patients with the BRAF V600E mutation reported an ORR of 41%, mPFS of 7.2 months, and mOS of 11.3 months [89]. Selumetinib, another MEK inhibitor, also showed potential in a phase II trial with an ORR of 12%, mPFS of 3.7 months, and mOS of 9.8 months [90]. Ulixertinib, an inhibitor of ERK 1/2, has demonstrated promising clinical efficacy in patients with MAPK-driven advanced tumors [91].

### 4.3. Targeted Therapies in NSCLC

#### 4.3.1. EGFR and MET Targeting Strategy

EGFR mutations, prevalent in approximately 15% of western and 40% of Asian NSCLC patient populations, are significant targets in oncology, with EGFR tyrosine kinase inhibitors (TKIs) dramatically altering the treatment landscape [92]. Osimertinib, a third-generation EGFR TKI, has become a first-line therapy for advanced NSCLC patients with sensitizing EGFR mutations, including the T790M mutation which confers resistance to earlier generation TKIs. However, resistance to osimertinib often develops after 1–2 years of treatment, leading to disease progression [93]. To overcome treatment resistance, various strategies are under investigation, including new EGFR TKIs effective against secondary EGFR alterations, and the dual-blockade approach using two EGFR inhibitors, or combining an EGFR TKI with another targeted agent [94].

Amivantamab, an EGFR-MET bispecific antibody, has been approved for NSCLC with EGFR exon 20 insertions [95]. In combination with lazertinib, a third-generation EGFR TKI, it targets EGFR at both the extracellular and catalytic domains, demonstrating synergistic inhibition of tumor growth. Early phase clinical trials in pre-treated patients have shown an encouraging ORR of 32%, with a combination treatment in a first-line setting achieving an ORR of 100% in treatment-naive patients [96]. MARIPOSA, a phase III randomized trial, is comparing this combination to osimertinib monotherapy as the first-line treatment for EGFR-mutant NSCLC, and has shown a PFS benefit, though OS data are pending [97].

#### 4.3.2. ALK

ALK rearrangements are found in approximately 5% of NSCLC patients, and currently, five ALK inhibitors (crizotinib, ceritinib, alectinib, brigatinib, and lorlatinib) are FDA-approved for this indication [98]. SAF-189s, a novel next-generation ALK inhibitor with CNS penetration, has shown the ability to overcome most known ALK resistance mutations in preclinical studies [99]. Early phase I/II trial results indicate promising mPFS and ORR improvements, especially in ALKi-naive patients [99]. Ongoing trials will help define the role of SAF-189s in ALK-altered NSCLC patients, especially those who have developed resistance to current ALK inhibitors.

#### 4.3.3. ROS1

ROS1 rearrangements, present in 1–2% of NSCLC cases, lead to auto-phosphorylation and activation of the MAPK pathway which drives cancer cell proliferation [98]. Crizotinib and entrectinib are FDA-approved treatments for ROS1-positive NSCLC, but resistance often develops. Repotrectinib, a newer inhibitor, showed an ORR of 40% to 67% in previously treated ROS1-positive NSCLC patients [100], while another novel ROS1 inhibitor taletrectinib achieved an ORR of 90% in TKI-naive patients [101]. These drugs received an FDA breakthrough therapy designation. NVL-520, a highly selective and brain-penetrant ROS1 inhibitor, demonstrated an ORR of 48% overall and 73% in patients with CNS metastasis in a phase I/II trial, showcasing its potential efficacy against ROS1 mutations, including the treatment-resistant G2032R mutation [102]. APG-2449, a TKI targeting ALK, ROS1, and focal adhesion kinase (FAK), has shown anti-tumor activity in preclinical models and is currently being tested in a phase I dose escalation and expansion trial that enrolled patients with ALK/ROS1 positive NSCLC [103]. At the recommended phase II dose, it demonstrated a good response rate, particularly in the first-line setting, with a favorable safety profile. Further large-scale clinical trials are necessary to confirm these findings. 

#### 4.3.4. RET

RET mutations, which are relatively uncommon in NSCLC, occurring in only 1–2% of cases, have been a focus of recent studies. Selpercatinib, a specific inhibitor targeting RET rearrangements, was assessed in the LIBRETTO-001 phase I–II study involving patients with RET-rearranged NSCLC. The study found an ORR of 64% among 105 patients who had previously received platinum-based treatments, and an impressive 85% ORR in 39 patients who had not undergone prior treatment. The median duration of response (mDoR) was recorded at 17.5 months for the previously treated group, while it was not reached (NR) in the treatment-naive group [104]. Another RET-selective inhibitor, pralsetinib, was examined in the ARROW study; it showed an ORR of 59% in 136 previously treated patients and 72% in 75 patients without prior treatment [105]. For this drug, the mDoR was not reported for the treatment-naive group and was 22.3 months for those previously treated. Notably, both drugs demonstrated high rates of intracranial response. Selpercatinib also showed its front-line superiority to chemoimmunotherapy in the LIBRETTO-431 trial [106]. Current EMA guidelines recommend the use of either selpercatinib or pralsetinib, specifically for patients who have not been treated with a RET inhibitor previously, for those patients with RET fusion-positive NSCLC [107].

#### 4.3.5. MET

MET mutations comprise altogether 8% of patients with NSCLC. Two type Ib MET inhibitors, capmatinib and tepotinib, received regulatory approvals for treating patients with MET exon 14 skipping mutations. In the GEOMETRY study, capmatinib demonstrated an ORR of 41% in 69 patients who had previously undergone treatment, and 68% in 28 patients without prior treatment; the mDoR was observed to be 9.7 months for the former group and 12.6 months for the latter [108]. In patients exhibiting high MET amplification (≥10 copies), capmatinib’s ORR was reported as 29% for those previously treated and 40% for treatment-naive patients [108]. In the VISION study, 152 patients with MET exon 14 skipping mutations treated with tepotinib found an ORR of 45% and a median duration of response of 11.1 months, with a mPFS of 8.9 months [109]. As per EMA guidelines, both capmatinib and tepotinib are recommended post-immunotherapy and/or platinum-based chemotherapy for patients with MET exon 14 skipping mutations [107]. While both drugs have approval for first-line treatment by the FDA, capmatinib is also recommended for patients with high MET amplification (≥10 GCN) following prior immunotherapy and/or platinum-based chemotherapy, although it lacks approval from both the EMA and FDA for this specific use.

#### 4.3.6. KRAS-G12C

KRAS mutations, present in 12% of NSCLC cases, are mutations which lack targeted therapies due to the absence of a deep binding pocket for inhibitors. They saw a breakthrough with the FDA approval of sotorasib, showing an ORR of 37.1% and mPFS of 6.8 months [110]. Adagrasib, another G12C inhibitor, displayed an ORR of 42.9% and mPFS of 8.5 months [111]. GDC-6036, a more selective KRAS G12C inhibitor, showed an ORR of 53% in a phase I trial [112]. D-1553, an oral inhibitor that selectively and irreversibly binds KRAS G12C, showed an ORR of 37.8% and a mPFS of 7.6 months [113]. 

#### 4.3.7. KRAS Non-G12C Targeting Strategy

For KRAS non-G12C mutations, different approaches are ongoing. Pan-RAS inhibitors are being developed, such as RMC-6236, a RAS-GTP inhibitor, which has shown early efficacy in KRAS G12D pancreatic cancer and G12V NSCLC [114]. Other options include the combination of VS-6766, a RAF/MEK clamp, and everolimus, an mTOR inhibitor. In a clinical trial involving patients with RAS or RAF mutant cancers, including KRAS mutant NSCLC, the combination showed a mPFS of 6.35 months with manageable toxicity, suggesting potential efficacy across different KRAS mutation variants [115]. The combination therapy is tolerable and shows promising preliminary clinical efficacy, warranting further trials to confirm its effectiveness in this subgroup of NSCLC patients.

### 4.4. Targeted Therapies in Melanoma

#### 4.4.1. BRAF/MEK Targeting Strategy

In cutaneous melanomas, the most common subtype, a high mutational burden, particularly in the BRAF gene, has led to the development of BRAF inhibitors (BRAFi) such as vemurafenib and dabrafenib. Specifically, these drugs are approved for treating metastatic melanomas with BRAF V600E and V600K mutations [116]. However, the development of resistance, primarily due to MAPK pathway reactivation, causes the effects of these BRAFi to be short-lived, with a mPFS limited to about 5–7 months [117,118]. To address this, combining BRAF inhibitors with MEK inhibitors like trametinib and cobimetinib has shown promise in prolonging the survival benefits by overcoming or delaying the onset of resistance and improving the mPFS to 11–12 months, as well as mitigating the unwanted side effect of paradoxical cutaneous lesions due to increased RAF activity and MAPK signaling in healthy skin [119,120]. MEK inhibitors such as binimetinib and pimasertib, have also been used to treat advanced NRAS-mutant melanoma. Although these inhibitors have shown effectiveness, the improvement in mPFS is typically around 3 months, indicating the rapid emergence of resistance pathways [121,122].

#### 4.4.2. KIT Targeting Strategy

KIT mutations are other frequently mutated genes in melanoma and offer another potential target for therapy. The KIT inhibitor imatinib has shown efficacy in providing a durable response in patients with KIT mutant metastatic melanoma, with a subset of patients showing a complete response of more than 1.5 years [123,124]. While not explicitly approved for melanoma, its effectiveness in a subset of melanoma patients underscores the potential of targeting KIT mutations in this cancer type. Ongoing studies are exploring the effectiveness of other KIT inhibitors, such as nilotinib and ripretinib, in treating melanoma [125,126].

### 4.5. Targeted Therapies in Colon Cancer

#### 4.5.1. EGFR Targeting Strategy

EGFR, part of the ErbB family of tyrosine kinase receptors, regulates crucial cellular processes and is implicated in cancer progression when overexpressed, as seen in many metastatic colorectal cancer (mCRC) cases. The overactivity of EGFR signalling is linked to poor patient outcomes, making it a significant target in mCRC therapy [127]. Cetuximab and panitumumab, both mAbs targeting EGFR, are approved for mCRC treatment in patients with non-mutated RAS genes. These treatments are specifically effective against mCRC without mutations in certain KRAS and NRAS codons. Cetuximab, a chimeric antibody, can stimulate antibody-dependent cell-mediated cytotoxicity (ADCC), thought to be primarily executed by natural killer cells and macrophages, which contributes to its therapeutic effect [128,129]. It displayed promise in a phase II trial, and further, the BOND trial showed cetuximab with irinotecan significantly improved OS to 22.9 months from 10.8 months in irinotecan-refractory patients [130,131]. Panitumumab, a fully human antibody, is less likely to induce immunogenic reactions compared to the chimeric cetuximab and has a lower risk of hypersensitivity reactions. However, it does not induce ADCC like cetuximab does [132]. The PRIME trial revealed that combining panitumumab with FOLFOX4 improved PFS (10.6 vs 8.6 months) and OS (22.9 months vs. 10.8 months) in mCRC patients compared to FOLFOX4 alone [133].

Both agents bind to domain III of EGFR and have been proven effective in wild-type RAS mCRC patients, showing comparable OS in the ASPECCT trial. Their distinct mechanisms of action mean that one can be effective following the failure of the other [134,135]. However, their use is restricted to wild-type RAS patients since those with RAS mutations generally do not benefit from EGFR-targeted therapies [136,137].

Interestingly, not all KRAS mutations confer resistance to EGFR-targeted treatments. Retrospective studies suggest that the KRAS codon G13D mutation may respond to cetuximab, showing intermediate responsiveness in cell lines and associated with longer PFS and OS compared to other KRAS mutations [138,139,140]. Further research, however, is required to fully understand the efficacy of EGFR-targeting strategies in patients with the G13D mutation compared to other KRAS mutations due to the small sample sizes in studies to date. Rechallenging with EGFR-targeting drugs is a promising strategy for mCRC patients who have previously responded to these treatments. The CRICKET trial demonstrated the benefits of rechallenging cetuximab in patients with RAS and BRAF-wt mCRC who developed resistance to initial cetuximab treatment, with a mPFS of 4.0 months compared to 1.9 months without rechallenge [141]. The CHRONOS trial further supported this approach, showing that a liquid biopsy-driven rechallenge with panitumumab could maximize therapeutic effects while minimizing adverse effects [142].

#### 4.5.2. BRAF Targeting Strategy

BRAF serves as an integral component of the RAS/Raf/MEK/ERK pathway and is recognized as a critical factor in oncogenesis [143]. V600E mutations in the BRAF gene occur in about 10% of mCRCs, which tend to have a reduced response to chemotherapy and a less favorable prognosis [144]. On the other hand, mCRCs with non-V600 mutations in the BRAF gene have been associated with a significantly enhanced OS [145]. Targeted monotherapy aimed at BRAF in mCRC patients has not been efficacious, prompting the investigation of combined therapeutic strategies in various clinical trials [146]. Encorafenib, which inhibits both the BRAF V600E mutant and the normal BRAF variant, demonstrates a more sustained pharmacodynamic response than its counterparts [147]. Data from the BEACON phase III study indicated that the use of encorafenib combined with cetuximab substantially increased the mOS to 8.4 months for 220 participants compared to 5.4 months for 221 participants receiving the conventional control treatment of cetuximab and chemotherapy. The treatment also elevated the confirmed response rate (RR) to 20% against the control’s 2% [148]. Additionally, in the BEACON trial, a regimen that included encorafenib, cetuximab, and the MEK inhibitor binimetinib resulted in a significant rise in mOS to 9.0 months for 224 subjects, as opposed to 5.4 months for 221 subjects in the control group, with a confirmed RR of 24% compared to 2% [148]. These outcomes led to the FDA’s approval of encorafenib in 2020 as a treatment option for mCRC with the BRAF V600E mutation after prior therapy.

#### 4.5.3. VEGF/VEGFR

The process of angiogenesis, essential for tumor growth, is influenced by the VEGF/VEGFR signaling pathway, crucial for the progression of cancer by activating vascular endothelial cells. In CRC treatments, especially for metastatic forms, VEGF/VEGFR-targeted strategies are employed, effective for various genetic mutations [149].

VEGF family proteins, including VEGF-A, B, C, D, and PlGF, interact with VEGFRs to initiate biological responses that promote angiogenesis and lymphangiogenesis. These interactions trigger tyrosine kinase activation and subsequent signaling pathways, critical for tumor angiogenesis and proliferation. Among these, VEGFR-1 and VEGFR-2 are particularly important therapeutic targets [150,151]. Bevacizumab is a mAb that inhibits VEGF-A from binding to VEGFR-1 and -2. Its FDA approval for mCRC was based on improved survival metrics: OS increased to 20.3 months from 15.6 months, PFS to 10.6 from 6.2 months, and RR to 44.8% from 34.8% with chemotherapy [14,152]. Subsequent studies have shown that bevacizumab extends survival after initial treatment progression, with OS benefits observed even in second-line treatments [153,154]. Despite these improvements, anti-EGFR therapies may be more effective in KRAS wild-type mCRC [155,156]. Aflibercept, with its high-affinity binding to VEGFs, is used with FOLFIRI following an oxaliplatin regimen failure. The VELOUR trial reported increased OS from 12.06 to 13.5 months and PFS from 4.67 to 6.9 months [157]. Ramucirumab, which inhibits VEGFR-2, has shown effectiveness when used with FOLFIRI for second-line mCRC treatment, improving OS to 13.3 months from 11.7 months and PFS to 5.7 from 4.5 months [158]. Regorafenib, a kinase inhibitor, has been approved for mCRC following the failure of other treatments, with clinical trials showing an increase in OS to 6.4 months from 5.0 months and PFS to 1.9 from 1.7 months [159]. Lastly, fruquintinib, a selective tyrosine kinase inhibitor, approved in China and on the FDA fast track, improved PFS and OS in mCRC patients in the FRESCO trials, with significant benefits for those beyond the third line of treatment [160].

#### 4.5.4. KRAS-G12C Targeting Strategy

KRASG12C mutations are seen in approximately 3% of mCRCs, accounting for 7% of all KRAS mutations [161]. KRASG12C mutations are associated with shorter durations of response to first-line chemotherapy compared to those with non-KRASG12C mutations [162]. Insofar, research has shown that monotherapy KRASG12C inhibitors such as sotorasib provide minimal benefits, such as seen in the CodeBreaK-100 study which assessed monotherapy sotorasib in chemorefractory mCRC with KRASG12C mutation (62 patients), with minimal responses seen (<10%) [163]. Recently, in the phase III trial CodeBreaK 300 investigating KRAS G12C inhibitor plus an EGFR inhibitor in patients with chemorefractory metastatic colorectal cancer, both doses of sotorasib in combination with panitumumab resulted in longer progression-free survival than standard treatment [164]. SHP2 inhibitors (e.g., TNO155) may provide an antitumor effect and overcome resistance seen in KRASG12C–mutant mCRC, which is also being investigated in combination with sotorasib in the CodeBreaK-101 study (NCT04185883). Adagrasib, another KRASG12C inhibitor, is also being investigated in combination with cetuximab in the randomized KRYSTAL-10 study in the second-line setting (NCT04793958).

#### 4.5.5. HER2

Amplification of HER2 is a rare condition in mCRC [161]. The HERACLES trial, which focused on mCRC patients with KRAS exon 2-wt and HER2-positive profiles, revealed HER2 blockade to have significant antitumor activity in select patients [165]. This study used a dual HER2 blockade approach, combining trastuzumab (an anti-HER2 mAb) with the tyrosine kinase inhibitor lapatinib. The results were promising, showing that 30% of patients experienced a partial or complete objective response rate (ORR), and an additional 44% achieved disease stability, indicating the potential of HER2 blockade therapies in treating mCRC.

### 4.6. Targeted Therapies in Gastric Cancer

#### 4.6.1. HER2 Targeting Strategy

In the evolving landscape of gastric cancer therapies, significant attention has been drawn to the overexpression of human epidermal growth factor receptor 2 (HER2), which is seen in about 10–20% of gastric cancer cases [166]. This overexpression is notably more prevalent in cancers at the proximal or gastroesophageal junction (OGJ) and in the intestinal subtype, as defined by Lauren [167]. For patients with gastric cancer showing HER2 overexpression (identified as either a HER2 immunohistochemistry (IHC) score of 3+ or a 2+ IHC score plus a positive fluorescence in situ hybridization (FISH) test), the addition of trastuzumab, an anti-HER2 antibody, to conventional platinum and fluoropyrimidine-based chemotherapy (ChT) has proven beneficial. This treatment strategy is the standard of care since the phase III ToGA study showed an OS benefit with a HR of 0.74 and low added toxicity [168]. Subsequent trials assessing the efficacy of second-line trastuzumab combinations, lapatinib, and trastuzumab emtansine in HER2-positive gastric cancer patients who had progressed on trastuzumab did not yield positive results [169]. In contrast, a recent phase II trial in Asia demonstrated a survival advantage with the HER2-targeting antibody drug conjugate, trastuzumab deruxtecan, over standard chemotherapy, a finding corroborated by global trials showing similar response rates in non-Asian populations [170]. 

#### 4.6.2. Claudin-18 Targeting Strategy

Another avenue being explored in gastric cancer treatment is targeting Claudin 18 isoform 2 (CLDN18.2), a component of tight junction proteins mainly found in stomach mucosa and maintained during malignant transformation [171]. The disruption of tight junctions in cancerous tissues may increase the exposure of CLDN18.2, presenting an attractive therapeutic target [172]. This has been substantiated by the FAST study which enrolled patients with advanced gastric/gastro-oesophageal junction and oesophageal adenocarcinoma exhibiting moderate-to-strong CLDN18.2 expression. The study compared the standard chemotherapy regimen EOX alone with EOX plus zolbetuximab, revealing that the addition of zolbetuximab improved PFS with a hazard ratio (HR) of 0.44 and OS with an HR of 0.55 [173]. The enhanced PFS and OS underline the therapeutic potential of targeting CLDN18.2 in gastric cancers, positioning zolbetuximab as a promising candidate for future treatment modalities in patients with CLDN18.2-positive gastric or gastroesophageal junction cancer.

#### 4.6.3. VEGF Targeting Strategies

The integration of ramucirumab, an antibody targeting the vascular endothelial growth factor receptor 2 (VEGFR2), with paclitaxel, has also shown promise, significantly enhancing OS (9.6 months vs. 7.4 months) in the phase III RAINBOW trial [174].

#### 4.6.4. Rare Occurrences: EFGR and MET Targeting Strategies

The correlation between EGFR overexpression and poor prognosis in advanced gastric cancer suggests the utility of EGFR-targeted therapies [175]. Trials like ATTAX3 phase II testing the addition of panitumumab to docetaxel, cisplatin, and fluoropyrimidine (DCF) in unselected patients showed disappointing results [176]. The mPFS for DCF alone was 6.9 months, compared to 6.0 months with panitumumab addition. Similarly, mOS was 11.7 months for DCF alone, and lower than 10 months with panitumumab. The REAL3 trial showed a slight OS improvement with panitumumab plus epirubicin, oxaliplatin, and capecitabine (EOC), but with increased toxicity [177]. The EXPAND trial reported no significant survival benefits with cetuximab [178]. These findings underscore the limited efficacy of EGFR inhibitors in unselected patient populations thus far, but potentially hold promise for the future.

**Figure 2:**
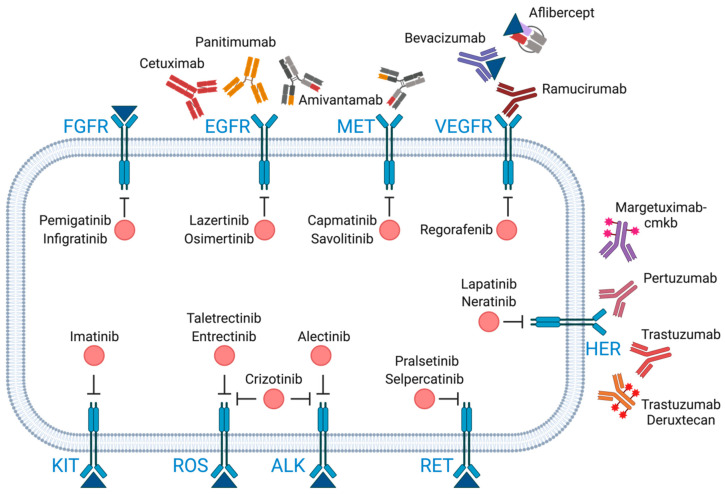
Therapeutic targets and corresponding drugs used in precision oncology. Receptor tyrosine kinases (RTKs) are crucial in cancer cell proliferation and survival. Each receptor is depicted on the cell membrane with associated therapeutic agents shown either as antibodies or small molecule inhibitors. **ROS:** ROS proto-oncogene 1; **FGFR:** fibroblast growth factor receptor; **EGFR:** epidermal growth factor receptor; **MET:** mesenchymal-epithelial transition factor; **VEGFR:** vascular endothelial growth factor receptor; **HER:** human epidermal growth factor receptor; **KIT:** Kit proto-oncogene receptor tyrosine kinase; **ALK:** anaplastic lymphoma kinase; **RET**: rearranged during transfection.

The development of MET inhibitors, including tyrosine kinase inhibitors (TKIs), multi-kinase inhibitors, and mAbs, is a significant area in gastric cancer drug development. Tivantinib (ARQ197) showed a DCR of 36.7% and mPFS of 43 days in phase I and II trials [179,180]. AMG 337 demonstrated an ORR of 18% with mPFS and OS of 3.4 and 7.9 months, respectively, in phase II [181]. Savolitinib in the VIKTORY trial reported a 50% ORR and met a 6-week PFS endpoint [182]. Crizotinib’s expanded cohort noted PFSs of 3.5 and 3.7 months in two patients [183]. Foretinib (GSK1363089) resulted in stable disease in 23% of patients [184]. Rilotumumab (AMG 102) improved mPFS in early-phase studies but failed in phase III [185]. Onartuzumab in the METGastric study showed no survival difference [186]. Emibetuzumab in phase II was well-tolerated with a PFS of 47% at 8 weeks [187] (Figure 2).

## 5. Discussion and Conclusions

While it is difficult to ascertain the impact of genomic research on routine medical practice, its direct influence certainly varies in academic versus non-academic environments. In the former, molecular tumor-boards will offer target-matched trials that may provide additional therapeutic options to patients. However, irrespective of the medical setting, genomics are undeniably transforming our knowledge of the biological aspects of almost every medical condition.

The landscape of cancer therapy is evolving from a focus on the tumor’s organ of origin to a more nuanced understanding of genomic alterations that transcend traditional categories. This evolution is exemplified by breakthroughs like that of KRAS G12C inhibitors, which target mutations once deemed “undruggable,” and the promise of future advancements in targeting TP53 mutations. Next-generation sequencing (NGS) has been pivotal in driving these advancements, enabling the identification of specific mutations and facilitating a shift towards gene-directed, personalized treatments (Table 1). Clinical trials are now increasingly grouping patients based on genetic profiles rather than cancer type, with trials like NCI-MATCH leading the way.

The development of targeted therapies has also seen the introduction of innovative methods such as proteolysis-targeting chimeras (PROTACs), which expand the range of druggable targets by directing the ubiquitin-proteasome system to degrade specific proteins. This approach, along with other emerging therapies like cancer vaccines, underscores the potential of precision medicine. Companion diagnostics, like FISH for HER2, are essential for validating NGS results, ensuring treatments are matched to the patient’s specific molecular changes with precision.

Despite the advances, challenges persist. The rapid pace of mutation detection has surpassed our ability to interpret the functional impact of each mutation, with some being incidental and others of uncertain significance. Innovations like computational modeling and CRISPR/Cas9 saturation mutagenesis are being developed to address these challenges. There remains significant progress to be made in reducing “off-target” effects and increasing specificity. In this purpose, sophisticated computational algorithms and bioinformatics tools are employed to predict potential off-target sites, aiding in the careful selection of target sequences with a minimal risk of unintended effects.

Moreover, the choice of therapy in the presence of multiple mutations requires nuanced clinical judgment.

Quality-anchored genomic testing, incorporating broad panels that detect markers like tumor mutational burden (TMB), homologous recombination deficiency (HRD), microsatellite instability (MSI), DNA, and RNA, enhances the sensitivity of detecting actionable mutations, especially fusions. However, the selection of panel size must balance thoroughness and practicality, focusing on genes with a high ESMO Scale for Clinical Actionability of molecular Targets (ESCAT) scores indicative of actionable mutations and prioritizing quality over quantity.

Economic considerations are also critical; cost-effective approaches are necessary regardless of panel size, covering targetable genes to ensure broad accessibility. The utility of liquid biopsies is context-dependent, solidified by evidence in conditions like EGFR lung cancer and PI3K and ESR1 breast cancer. Understanding allelic frequencies and validated thresholds from registration trials is crucial, particularly for companion diagnostics.

The disparity between routine practice and specialized cancer center approaches highlights the importance of access to agnostic therapies. Currently, lung cancer is the primary area where NGS offers a cost-effective solution. The future success of targeted therapies will rely on advances in targeting strategies and a deeper understanding of patient responses and resistance mechanisms.

Lastly, molecular tumor boards are critical, utilizing NGS as a platform to integrate patients into clinical trials, enhancing the potential for tailored treatment strategies and improved patient outcomes. 

## Figures and Tables

**Table 1:** Specific mutations and targeted therapies.

Tumor	Molecular Alteration	Targeted Options	Validation Trial	Median OS (Months)	Median PFS (Months)	FDA Approval	ESCAT	Reference
**NSCLC**	**EGFR ex 19 et 21**	Osimertinib	FLAURA	-	8.7	18 April 2018	IA	[188]
		Lazertinib	LASER301	-	10.9	-		[189]
	**EGFR ex20ins**	Amivantamab + Lazertinib	MARIPOSA	-	4.5	-	IB	[97]
	**ALK**	Alectinib	ALEX	-	23.9	6 November 2017	IA	[190]
	**ROS1**	Crizotinib	PROFILE 1001	51.4	19.3	11 March 2016	IB	[191]
		Entrectinib	ALKA-372-001, STARTRK-1, and STARTRK-2	47.8	15.7	15 August 2019		[192]
	**MET exon 14**	Capmatinib	GEOMETRY	20.8	10.8	10 August 2022	IB	[108]
		Tepotinib	VISION	19.6	11.2	15 February 2024		[193]
	**KRAS G12C**	Sotorasib	CodeBreaK100	12.5	6.3	28 May 2021	IB	[194]
		Adagrasib	KRYSTAL-12	-	5.49	12 December 2022		[195]
	**RET**	Selpercatinib	LIBRETTO001	-	16.5	21 September 2022	IB	[196]
**Breast cancer**	**BRCA**	Olaparib	OlympiAD	-	7	12 January 2018	IA	[63]
	**HER2**	Pertuzumab+ trastuzumab + docetaxel	CLEOPATRA	56.5	18.7	8 June 2012		[47]
		Trastuzumab deruxtecan	DESTINY	23.4	9.9	20 December 2019		[197]
		TDM1	EMILIA1	30.9	9.6	22 February 2013		[48]
	**CDK**	Abemaciclib	MONARCH 3	66.8	29	28 September 2017	IA	[198]
		Ribociclib	MONALEESA 2	63.9	25.3	18 July 2018		[59]
		Palbociclib	PALOMA	53.9	24.8	19 February 2016		[199]
	**PIK3CA**	Alpelisib	SOLAR-1	39.3	11.1	24 May 2019	IA	[61]
	**mTor**	Everolimus	BOLERO-21	31	7.8	20 June 2012	IA	[200]
**Cholangiocarcinoma**	**IDH**	Ivosidenib	ClarlDHy	10.3	6.9	25 August 2021	IA	[87]
	**FGFR2 mutation**	Pemigatinib	FIGHT-202	17.5	7.0	17 April 2020	IB	[201]
		Infigratinib	PROOF 301	-	7.4	28 May 2021		[202]
**Colon**	**EFGR**	Cetuximab	BOND	8.6	4.1	12 February 2004	IA	[131]
		Panitumumab (+FOLFOX)	PRIME 3	23.9	9.6	1 October 2006		[203]
	**VEGF**	Bevacizumab (+capecitabine)	AVEX	-	9.1	26 February 2004	IA	[204]
		Aflibercept (+FOLFIRI)	VELOUR	13.5	6.9	3 August 2012		[157]
		Regorafenib	CORRECT	6.4	1.9	27 September 2012		[159]
		Ramucirumab (+FOLFIRI)	RAISE	13.3	5.7	24 April 2015		[158]
	**BRAF/MEK**	Encorafenib + Cetuximab	BEACON	9.0	4.3	8 April 2020	IA	[148]
**Melanoma**	**BRAF/MEK**	Dabrafenib-trametinib	COMBI-D/COMBI-V (pooled analysis)	25.9	11.1	9 January 2014	IA	[205]
						25 November 2015		
		Vemurafenib-Cobimetinib	coBRIM	22.5	12.6			[206]
		Encorafenib-binimetinib	COLOMBUS	36.8	14.9	27 June 2018		[28]
	**c-KIT**	Imatinib	Hodi et al.	12.5 months	3.7 months	-	N/A	[123]
**Gastric**	**HER2**	Trastuzumab (+chemotherapy)	ToGa	13.8	6.7	21 October 2010	IA	[168]
	**VEGF**	Ramucirumab	REGARD	5.2	2.1	21 April 2014	N/A	[207]
	**CLDN18.2**	Zolbetuximab	FAST	18.23	10.61	-	N/A	[208]

List of Abbreviations: OS, overall survival; PFS, progression-free survival; ESCAT, ESMO Scale for Clinical Actionability of Molecular Targets; DoR, duration of response; ORR, overall response rate; TTP, time to progression; HR, hazard ratio; NSCLC, non-small cell lung cancer; EGFR, epidermal growth factor receptor; ALK, anaplastic lymphoma kinase; ROS1, c-ros oncogene 1; MET, MET proto-oncogene, receptor tyrosine kinase; KRAS, Kirsten rat sarcoma viral oncogene homolog; RET, RET proto-oncogene; BRCA, breast cancer gene; HER2, human epidermal growth factor receptor 2; CDK, cyclin-dependent kinase; PIK3CA, phosphatidylinositol-4,5-bisphosphate 3-kinase catalytic subunit alpha; mTOR, mammalian target of rapamycin; IDH1, isocitrate dehydrogenase 1; FGFR2, fibroblast growth factor receptor 2; GIST, gastrointestinal stromal tumor; KIT, KIT proto-oncogene receptor tyrosine kinase; PDGFR, platelet-derived growth factor receptor; VEGF, vascular endothelial growth factor; BRAF, v-Raf murine sarcoma viral oncogene homolog B; CLDN18.2, Claudin 18.2.
